# Evaluation of toxic effects of dapagliflozin on reproductive system in diabetic rats

**DOI:** 10.55730/1300-0144.5443

**Published:** 2022-03-12

**Authors:** Bahar ULUS KARACA, Tuğçe BORAN, Ayça KARAGÖZ KÖROĞLU, Feriha ERCAN, Gül ÖZHAN

**Affiliations:** 1 Department of Pharmaceutical Toxicology, Faculty of Pharmacy, İstanbul University, İstanbul, Turkey; 2 Department of Histology, Faculty of Medicine, Marmara University, İstanbul, Turkey

**Keywords:** Type-2 diabetes, dapagliflozin, male reproductive system, oxidative damage

## Abstract

**Background/aim:**

Dapagliflozin (DAPA), sodium-glucose cotransporter 2 (SGLT2) inhibitor, is an insulin-independent antidiabetic drug used to control hyperglycaemia by promoting glucose excretion from the kidney. Its adverse effects include orthostatic hypotension, dehydration and urinary tract and genital infections caused by glycosuria. DAPA is subjected to constant additional monitoring, as drug-related adverse reactions are frequently updated in line with the results of case studies, clinical trials and in vivo studies. Some antidiabetic drugs have shown potential harmful effects on the male reproductive system; however, the effects of DAPA have not been sufficiently studied in this capacity. Aiming to fill this gap in the literature, the present work investigates the toxic effects of DAPA on the male reproductive system.

**Materials and methods:**

Diabetes was induced using streptozotocin (STZ) in adult male Sprague-Dawley (SD) rats. DAPA (10 mg/kg) was administered by gavage to the diabetic rats over 28 days, after which the animals were sacrificed. The biochemical, morphological and histological examinations were performed on testicle, sperm and plasma samples.

**Results:**

As a result of this study, we observed reproductive system damage in the form of induction of apoptosis in the seminiferous tubules, changes in testis and sperm parameters and oxidative damage, alongside the development of diabetes in test animals. With the exception of sperm morphological damage, the changes observed in diabetic animals treated with DAPA were similar to those of the control group. Improvements were observed in histological, hormonal and proliferative parameters in the DAPA group compared to the DC group.

**Conclusion:**

Even if DAPA is found to have antioxidant effects, it may raise abnormal sperm counts through a mechanism completely independent of these effects and thus may not have a significant toxic effect on the male reproductive system.

## 1. Introduction

Since the 10th century, diabetes has been associated with sexual dysfunction. Avicenna described this a “collapse of sexual function” as a special complication of diabetes. Diabetes causes significant changes on the various structures and mechanisms of the male reproductive system through endocrine disorders, neuropathy and increased oxidative stress. Diabetic infertility has been shown to affect 15% of couples, and diabetes is becoming an increasingly complicated health problem worldwide. It is important to control blood glucose levels in diabetes patients because damage is caused by glucose toxicity due to hyperglycaemia [1,2].

DAPA is an insulin-independent antidiabetic drug that acts as a SGLT2 inhibitor and aims to control hyperglycaemia via providing glucose excretion from the kidney through a mechanism different to that of other antidiabetic drug groups. The major adverse effects of SGLT2 inhibitors are hypoglycaemia, orthostatic hypotension, dehydration, urinary tract infection and genital infections caused by glycosuria. These infections have been shown to progress to serious problems such as acute kidney injury, urosepsis, pyelonephritis, bladder cancer, vulvovaginitis and balanitis^1^. In addition, DAPA has been associated with limb amputation, Fournier gangrene, diabetic ketoacidosis, metabolic bone disease and increased haematopoiesis [3]. Adverse effects related to DAPA use are frequently updated and it is included in the FDA’s watch list^2^.

As well as the genital infections caused by DAPA, its use can lead to loss of libido and sexual motivation in men [4,5]. The literature lacks sufficient information about the effects of DAPA on the male reproductive system, thus leading us to investigate these potential effects and mechanisms in the case of repeated exposure.

## 2. Material and methods

### 2.1. Animals and study design

Twenty-one (10–12 weeks old) male SD rats (250–300 g) were obtained from the experimental animal application and research centre of Acıbadem University, Turkey. Four or five animals were housed per standard polystyrene cage and provided with food and water ad libitum. The rats were maintained at 21–23 °C and humidity (55 ± 5%) in a light-dark cycle in the Laboratory Animal Facility unit of İstanbul University. Our experimental procedures were approved by the İstanbul University Local Ethics Committee for Experimental Animals (IUHADYEK 02.03.2018-2818/24) and all the work was carried out in accordance with ethics committee principles. Following the method of Furman (2015) with minor revisions, a type 2 diabetes (T2DM) model was created with a high-fat diet (35% fat, 26% protein and 26% carbohydrate) for two weeks then application (i.p. injection; 35 mg/kg in 0.1 M citrate buffer, pH 4.5) of a single dose of STZ (Sigma-Aldrich, Saint Louis, MO, USA). Diabetes was verified by measuring blood glucose levels from the tail blood of the animals with a glucose analyser device (Vivacheck, Biotech Inc., China). The animals with a nonfasting blood glucose level >270 mg/dL were included in the experiment as the diabetic group (n = 16) [6]. The diabetic animals were randomly divided into two groups and diabetic control (DC) (n = 8) and DAPA (n = 8) groups were formed. While 10 mg of DAPA was administered to the DAPA group for 28 days, the healthy control (C) and diabetic control groups were given water and 0.5% methylcellulose respectively by oral gavage.

The area under the plasma drug concentration-time curve (AUC) at 10 mg/kg DAPA, which showed the maximum glucose-lowering effect in SD rats, was 130-fold greater than for humans at the same dose [7,8]. Accordingly, we used 10 mg/kg dose of DAPA in 0.5% methylcellulose for the experiment.

On the 28th day, the rats were sacrificed by taking heart blood under inhalation anaesthesia. Blood samples were placed in EDTA-treated tubes for biochemical analysis. The left testicle and epididymis were fixed in PBS (1X) for biochemical analysis, and the right testicle and epididymis were fixed in neutral buffered formalin (10%) and stored at room temperature for histological studies.

### 2.2. Body and organ weights

The general condition of the animals, mortality and clinical signs e.g., movements, posture, appearance and locomotor activity were monitored daily, and body weight was recorded 3 times a week throughout the study and on the day of sacrification. The testicles were removed and weighed immediately after sacrification. The somatic index was calculated by dividing organ weight by body weight.

### 2.3. Sperm count and morphology

The epididymis was cleaned from the surrounding tissues and cut into small pieces with scissors in 1 mL of DMEM-F12 medium with PBS to release sperm. The suspension was centrifuged at 2230 rpm for 3 min. The supernatant (100 μL) was added to trypan blue (1:1, v/v). The mixture was mounted on a Thoma slide and sperm was counted under Leica microscope (Leica Microsystems Co., Wetzlar, Germany) at 20X magnification.

To investigate sperm morphology, one drop of the epididymis homogenate was spread on the slide. After drying at room temperature, the slides were stained with a Diff Quick staining kit (ADR, İstanbul, Turkey). Three slides were prepared from each sample and 200 sperms were evaluated from each slide. For morphological examination, sperms were classified as normal, headless, flattened head, pinhead, tailless, broken tail, broken neck and multiple anomaly sperm^3^ [9].

### 2.4. Biochemical and histological examination

Blood samples in EDTA-treated tubes were centrifuged at 3000 rpm for 20 min and stored at –20 °C. The testicular tissues in PBS (1:5, w/v) were stored at –80 °C, and homogenized on ice at 4 °C on the day processed. Testosterone, GSH and MDA levels were measured in plasma and tissue samples using enzyme-linked immunosorbent assay (ELISA) commercial kits (Elabscience, Wuhan, China; SunRed, Shanghai, China) according to the manufacturer’s instructions.

Left testicles were fixed in a neutral buffered formalin solution (10%) for 72 h then dehydrated by passing through alcohol series and embedded in paraffin [10,11]. Thick sections (4 μm) were taken from paraffin blocks and stained with Haematoxylin and Eosin (H&E) for histopathological semiquantitative analysis. The first seminiferous tubule was randomly selected and others were selected in clockwise fashion. Twenty seminiferous tubules were examined from each section with the modified Johnson scoring method (Table 1) [12] at 200X magnification. Seminiferous tubule basement membranes were stained with periodic acid-Schiff (PAS) dye for evaluation.

Endogenous peroxidase activity was suppressed by immersion in hydrogen peroxide solution (3%). To ensure antigen recovery, sections were exposed to microwave treatment in heated citrate buffer (pH: 6) for 20 min. After washing with PBS, the slides were incubated overnight at 4 °C with the PCNA primary antibody (Thermo Scientific, Massachusetts, USA). They were again washed with PBS then stained with UltraTek HRP Anti-Polyvalent Staining System (Scytek, Utah, USA) for 20 min according to manufacturer’s protocol. Slides were washed with PBS, 3,3-diaminobenzidine tetrahydrochloride dihydrate (DAB) (Abcam, Cambridge, UK) was applied and the colour reaction was observed under the microscope. After staining was checked, sections were counterstained with Mayer haematoxylin (Millipore, Massachusetts, USA) and mounted with entellan, then examined under a light microscope. Twenty seminiferous tubules were examined in tissue sections. The proliferation index was calculated for each seminiferous tubule by dividing the number of PCNA-positive cells by the total number of cells.

TUNEL assay was performed with ApopTag Plus Peroxidase In Situ Apoptosis Kit (Millipore, Massachusetts, USA) according to manufacturer’s protocol. Twenty randomly selected seminiferous tubules were examined from the tissue sections. The seminiferous tubules with a TUNEL^+^ cell count of 3 or more were counted. The apoptotic index was calculated by dividing the number of seminiferous tubules containing 3 or more TUNEL^+^ cells by the total number of seminiferous tubules.

### 2.5. Statistical analysis

Power analysis was performed with G*Power software (version 3.1.9.7). The effect size of the study was 0.9804 (n = 21). Results are given as values ± standard error of the means (SEM). In statistical analysis, Statistical Package for Social Sciences (SPSS, v.20, IBM Corporation, Armonk, NY, USA) was used. The homogeneity of variances was assessed using Levene’s test and the distribution of normality was evaluated by the Shapiro–Wilk test (*p* > 0.05). Statistical significance of differences between groups was validated with one-way analysis of variance (ANOVA) followed by posthoc Tukey test for normally distributed data. Kruskal–Wallis test was used for the data which are not normally distributed. Values of *p* < 0.05 was considered to be significant for all assays.

## 3. Results

Monitoring the animals daily, we observed a decrease in movement and an increase in blood glucose levels, water consumption, diuresis and fatigue in diabetic animals. The mean weights of rats are given in Table 2. While normal animals gained weight day by day, we observed weight loss as a result of STZ application in diabetic animals. There was a significant decrease in weights of the DC group (1.84-fold) compared to the gains observed in group C (*p*: 0.020). While there was no difference between testicular weights, there was a significant increase in the somatic index values of diabetic groups compared to C (*p* ≤ 0.007).

We found a significant decrease in the sperm counts of the DC and DAPA groups compared to the control (p < 0.002). After DAPA application, the sperm count decrease associated with diabetes was less pronounced in the DAPA group compared to the DC group (*p* < 0.001) (Table 3). The number of sperms with anomalies increased by 25.12% in the DC group, compared to the C group, and by 1.3-fold in the DAPA group (*p*: 0.003). The number of anomaly sperms showed a significant increase in the DAPA compared to the DC group (0.9-fold) (*p*: 0.003). In DAPA, there was an increase especially in detached head, bent tail, coiled tail and multiple abnormalities-type morphological disorders.

As it can be seen in Table 4, while plasma and tissue testosterone levels decreased in both diabetic groups, they were higher in the DAPA group compared to the DC group. Testicular testosterone levels decreased significantly in the DC group (*p*: 0.002) compared to the C group, while there was a significant increase in the DAPA compared to the DC group (*p*: 0.006).

Similar to testosterone levels, plasma and testicular GSH levels decreased in both diabetic groups, butwere higher in the DAPA group than in DC group. Testicular GSH levels decreased significantly in the DC group compared to the C group (*p*: 0.009) and were significantly higher in DAPA than in the DC group (*p*: 0.008). Compared to the C group, the increase in plasma MDA levels in the diabetic groups was statistically significant (*p* ≤ 0.046); however, the increase was lower in the DAPA group (21.78%). This significant difference in plasma results was not observed in tissue MDA levels.

**Figure 1:**
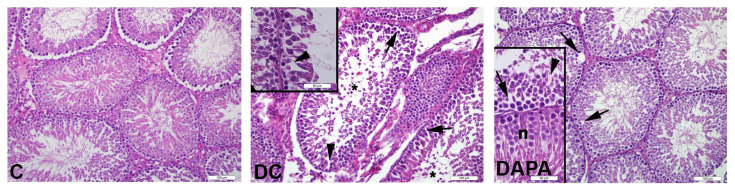
Histopathological imaging of the seminiferous tubules of experimental animals with H&E stain. Representative photomicrographs of experimental groups. Normal testicular morphology with regular spermatogenic cells and seminiferous tubules in group C; decrease in spermatogenic germ cell count in DC group (arrowhead), large vacuole formation between cells (arrow), immature cell debris in the lumen (*); in the DAPA group, seminiferous tubules with normal morphology (n) as well as seminiferous tubules in which large vacuole formations (arrow) between cells are considerably reduced.

**Figure 2:**
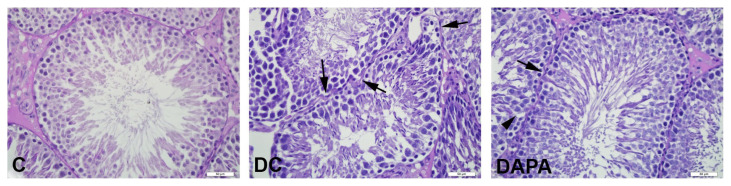
Histopathological imaging of the seminiferous tubules of experimental animals with PAS staining. Representative photomicrographs of experimental groups. Seminiferous tubules with regular and PAS-positive basement membrane in group C; seminiferous tubules with irregular and undulating basement membrane (arrow) structure and irregular PAS positive reaction in DC group; in the DAPA group, seminiferous tubules with a fairly regular basement membrane structure showing positive reaction to PAS.

Group C exhibited normal testicular morphology with regular spermatogenic cells and seminiferous tubules and regular basement membrane (score 9.1). Spermatagonia, primary and secondary spermatocytes, early spermatids and spermatozoa were observed with regular morphology in most seminiferous tubules. In DC group (score 6.7), a large number of seminiferous tubules showed decreased numbers of spermatogenic germ cells, large vacuole formation between the cells and immature cell debris in the lumen. In addition, many seminiferous tubules in this group had irregular and undulating basement membrane structures. In the seminiferous tubules of the DAPA group (score 7.3), we observed a considerable reduction in the formation of large vacuoles between cells and undulating basement membrane structure, as well as increasing numbers of spermatogenic germ cells and normal seminiferous tubules (Figures 1 and 2). There was a significant decrease in seminiferous tubule scores in DC and DAPA groups compared to C (*p*: 0.000). Similarly, the DAPA group’s seminiferous tubule score was significantly higher than DC group (*p*: 0.004) (Figure 1, Table 5).

**Figure 3:**
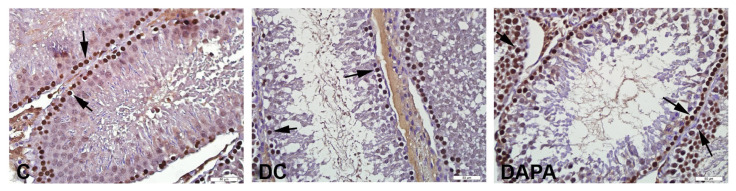
Imaging of the seminiferous tubules of experimental animals by PCNA immunohistochemistry. Representative photomicrographs showing PCNA positive cells belonging to experimental groups. Multiple PCNA positive cells in seminiferous tubules in group C (arrow); decreased PCNA positive cells (arrow) in the DC group, increased PCNA positive cells (arrow) in the DAPA group.

PCNA^+^ cells were seen in the seminiferous tubules of all groups (Figure 3). Compared to the C group, a significant decrease was detected in the proliferation index of the DC and DAPA groups (*p* ≤ 0.022). The proliferation index decrease associated with diabetes was less pronounced in the DAPA group compared to DC group (*p <* 0.001) (Table 5).

**Figure 4:**
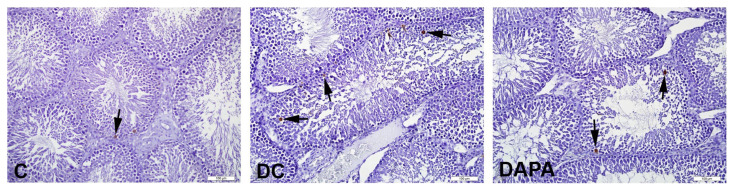
TUNEL staining in seminiferous tubule tissue of experimental animals. Representative photomicrographs showing TUNEL+ cells belonging to experimental groups. In group C, a small number of TUNEL^+^ cells (arrow) in the seminiferous tubules, an increase in the number of seminiferous tubules containing 3 and more TUNEL+ cells in the DC group, and a decrease in the number of seminiferous tubules containing 3 and more TUNEL+ cells (arrow) in the DAPA group.

TUNEL^+^ cells were seen in the seminiferous tubules of all groups (Figure 4). Apoptotic index values based on TUNEL^+^ cell count, increased significantly in the DC group (*p*: 0.004) compared to the C group, while there was a significant decrease in the DAPA compared to the DC group (*p*: 0.001) (Table 5).

## 4. Discussion

In order to investigate the male reproductive toxicity of DAPA, STZ-induced diabetic male SD rats were orally exposed to 10 mg/kg of DAPA once daily for 28 days. We then evaluated the testicular weight; testicular somatic index; plasma and testicular testosterone content; GSH and MDA levels; sperm count; sperm morphology; TUNEL apoptotic cell count and PCNA immunohistochemistry, as well as the macroscopic, microscopic and histopathological characteristics of testicular tissue.

The STZ-induced diabetes model has been reported to cause significant weight loss in animals [13–16]. Similarly, we observed significant weight loss in the DC group that received STZ compared to the C group (1.84-fold). The body weight of the DAPA group was higher than the DC group but not significantly so, and there was no notable change in the weight of the DAPA group between the first and last days in our study. The weight loss caused by STZ may have been mitigated by the hyperphagia observed in the DAPA group [7,16,17].

STZ-induced diabetes has been reported to cause a decrease in testicular weights and testicular somatic index [18,19]. In the present study, we observed no significant difference in testicular weights of the diabetes groups compared to the C group, corroborating the results of Abbasi et al. [20]. However, the testicular somatic index value increased significantly in DC and DAPA groups compared to group C (≤ 22.44%). The increase might be explained by the notable weight loss observed in those animals [14,21].

Diabetes is known to affect spermatogenesis, decrease sperm count and alter sperm morphology [14,18,22,23]. In this study, we observed a significant decrease in the sperm count of diabetic groups compared to the C group (≥ 29.66%). The DAPA group count was significantly higher than DC group (1.3-fold), a result which could be related to the antioxidant effect of DAPA [24,25].

Abnormal sperm count increased in both diabetic groups, but the difference between the DC group and C group was not significant. Similarly, some studies have observed no significant difference between the sperm morphologies of diabetic patients and healthy people [26,27]. In the present study, the number of sperm with impaired morphology was higher in the DAPA group than the DC group. In other words, DAPA was observed to cause damage to sperm morphology independent of the effect of diabetes. While the improvements occurring in all testicular and sperm parameters are thought to result from antioxidant effects, deterioration in sperm morphology suggests that the damage may be caused by another mechanism. Similarly, Venkatesh et al. examined the relationship between sperm morphology and oxidative stress, and observed there is no correlation between them [28]. Sperm formation and differentiation is a multistage process in which many factors play a role, and further studies are needed to understand how DAPA causes morphological damage, and at which stage.

Studies have reported that diabetes generally affects the male reproductive system through oxidative damage and significant improvements have been achieved with antioxidant therapy in many animal and clinical studies [18,29]. Ding et al. reported that NO and reactive oxygen species reduced fertilization rates and sperm quality, causing damage to the testicle and resulting in apoptosis and testicular atrophy [22]. Shrilatha [23] found that diabetes caused oxidative damage and raised MDA levels in the testes of diabetic mice, leading to increased levels of testicular GSH and other enzymes (PK, GSH-Px, GST and SOD) that result from oxidative damage. Similarly, plasma and testicular GSH levels decreased while MDA levels increased in diabetic groups in our study. Compared to the C group, the decrease in testicular GSH levels of the DC group (34.4%) and the increase in plasma MDA levels of the DC and DAPA group (≥ 1.82-fold) were found to be statistically significant. In the DAPA group, testicular GSH depletion was lower (47.54%), compared to DC group. The results may be due to the antioxidant effect of DAPA [24,25].

Diabetes is known to disturb the secretion of many hormones effective in the male reproductive system. Insulin has a stimulating effect on testosterone production and a decrease in testosterone levels is observed in its deficiency [23,30,31]. Decreased testosterone levels have been reported in STZ-induced diabetic animals [14,22]. In our study, as with other oxidation-affected parameters, testicular testosterone levels decreased significantly in DC group compared to the control (31.68%), whereas there was a significant increase in testosterone in the DAPA group compared to the DC group (28.94%). Although a similar situation was observed in plasma testosterone levels, the differences were not statistically significant.

Diabetes also causes histological damage, including decreased germ cell count, atrophy of seminiferous tubules, disruption of seminiferous tubules and cells, and accumulation of immature and irregular cells in the cell lumen [14,18,19]. Contrary to other studies, Navarro-Casado et al. reported no change in testicular histology associated with STZ-induced diabetes in 45 and 60 mg/kg single doses [31]. Similar to the studies mentioned above, our work observed a decrease in the number of spermatogenic germ cells, large vacuole formation between cells and immature cell debris in the lumen of many seminiferous tubules in DC and DAPA groups, compared to the C group. In the DAPA group, large vacuole formation between cells in the seminiferous tubules decreased significantly, while numbers of spermatogenic germ cells and normal seminiferous tubules increased, compared to the DC group.

Diabetes is known to promote apoptotic death and inhibit cell proliferation in germ cells such as spermatogonia and spermatocytes [21,32–34]. In parallel with the existing literature, we observed that apoptotic cells increased among the spermatogonia and primary spermatocytes of diabetic groups. The DAPA group, on the other hand, saw significantly lower rates of apoptotic death and apoptotic index alongside increased cell proliferation and proliferation index.

In conclusion, the formation of diabetes in animals leads to the induction of apoptosis and oxidative damage in the seminiferous tubules, while damage to the reproductive system occurs through an observable change in testicular tissue and sperm parameters. The damage was less pronounced in diabetic animals treated with DAPA, which had results similar to the control in all areas investigated except for sperm damage. It is thought that the improvements may be due to the glucose lowering or antioxidant effects of DAPA. However, DAPA caused to increase the number of morphologically damaged sperm, and further studies are needed to understand the underlying mechanism for sperm damage. The present work is the first to study the effects of DAPA on the male reproductive system. DAPA-induced healthy animal group could not be evaluated because there was no approval in ethical committee, which is the limitation of the study.

## Figures and Tables

**Table 1:** Modified Johnson scoring method [15].

Score	Histological findings
10	Full spermatogenesis
9	Slightly impaired spermatogenesis, many late spermatids, disorganized epithelium
8	Less than five spermatozoa per tubule, few late spermatids
7	No spermatozoa, no late spermatids, many early spermatids
6	No spermatozoa, no late spermatids, few early spermatids
5	No spermatozoa or spermatids, many spermatocytes
4	No spermatozoa or spermatids, few spermatocytes
3	Spermatogonia only
2	No germinal cells, Sertoli cells only
1	No seminiferous epithelium

**Table 2:** Body weights on the 1st and 28th days testicular weights and testicular somatic index of the rats.

	C	DC	DAPA
Body weights (g), 1st day	355.84 ± 14.75	314.52 ± 10.25	318.1 ± 15.57
Body weights (g) 28th day	377.00 ± 15.18	296.85 ± 16.27*	330.55 ± 15.94
Testes weights (g)	1.507 ± 0.045	1.52 ± 0.025	1.612 ± 0.063
Somatic index (testis)	0.401 ± 0.011	0.514 ± 0.022*	0.491 ± 0.016*

Data were shown as mean ± SEM.

* significant difference compared to C group (p < 0.05).

**Table 3:** Sperm count and morphological examination results of animals.

Sperm count (×10^6^/mL)			
	C	DC	DAPA
Sperm count	87.22 ± 6.65	26.69 ± 2.96*	61.35 ± 3.82*^a^
**Sperm morphology (in 200 sperm) (mean ± SEM)**			
	**C**	**DC**	**DAPA**
	**Normal sperm count (mean ± SEM)**		
Normal	179.7 ± 1.2	174.7 ± 3.7	154.2 ± 4.7*^a^
	**Sperm count with abnormalities (mean ± SEM)**		
Headless sperm	3.3 ± 0.6	5.3 ± 1.5	6.7 ± 1.0
Detached head	1.5 ± 0.7	4.7 ± 1.6*	8.8 ± 3.11*^a^
Flattened head	2.8 ± 0.7	1.3 ± 0.2	2.3 ± 0.6
Pinhead	1.0 ± 0.8	0.3 ± 0.2	0.5 ± 0.2
Bent neck	1.4 ± 0.3	2.8 ± 0.5	1.6 ± 0.3
Bent tail	6.3 ± 1.0	8.4 ± 1.3	14.7 ± 2.0*
Coiled tail	3.2 ± 0.8	1.6 ± 0.5	7.2 ± 1.5^a^
Multiple abnormalities	0.7 ± 0.3	0.9 ± 0.2	4.0 ± 1.0*
Total sperm count with abnormalities	20.3 ± 1.2	25.4 ± 3.7	47.5 ± 5.8*^a^
Percentage of the sperm with abnormalities (%)	9.9 ± 0.6	12.7 ± 1.8	23.8 ± 2.9*^a^
Percentage of the normal sperm (%)	89.9 ± 0.6	87.3 ± 1.8	77.1 ± 2.4

Data were shown as mean ± SEM.

* significant difference compared to C group (p < 0.05).

a significant difference compared to DC group (p < 0.05).

**Table 4:** Testosterone, GSH and MDA levels of animals in plasma and testes tissue.

Plasma levels (mean ± SEM)			
	C	DC	DAPA
Testosterone (ng/mL)	0.656 ± 0.033	0.593 ± 0.009	0.652 ± 0.019
GSH (μmol/mg protein)	0.054 ± 0.004	0.033 ± 0.003	0.052 ± 0.008
MDA (nmol/mg protein)	0.028 ± 0.005	0.101 ± 0.011*	0.079 ± 0.016*
**Tissue levels (mean ± SEM)**			
Testosterone (ng/mL)	2.989 ± 0.259	2.042 ± 0.022*	2.633 ± 0.122^a^
GSH (μmol/mg protein)	0.093 ± 0.007	0.061 ± 0.005*	0.090 ± 0.007^a^
MDA (nmol/mg protein)	0.075 ± 0.008	0.115 ± 0.005	0.112 ± 0.015

Data were shown as mean ± SEM.

* significant difference compared to C group (p < 0.05).

a significant difference compared to DC group (p < 0.05).

**Table 5:** Modified Johnson scoring, proliferation index and apoptotic index in the testes tissue.

	C	DC	DAPA
Seminiferous tubule score	9.05 ± 0.09	6.71 ± 0.15*	7.33 ± 0.11*^a^
Proliferation index	27.45 ± 0.58	20.36 ± 0.41*	25.58 ± 0.33* a
Apoptotic index (%)	3 ± 1.22	11.25 ± 1.57*	3.13 ± 1.32 a

Data were shown as mean ± SEM.

* significant difference compared to C group (p < 0.05).

a significant difference compared to DC group (p < 0.05).

## Footnotes

1 Food and Drug Administration (FDA) (2013). Risk Assessment and risk mitigation review(s). Application number: 202293Orig1s000 [online]. Website https://www.accessdata.fda.gov/drugsatfda_docs/nda/2014/202293Orig1s000RiskR.pdf [accessed 15 December 2019].

2 Food and Drug Administration (FDA) (2017). New FDA watch list flags more drugs [online]. Website https://www.medscape.com/vewartcle/882425 [accessed 12 December 2019].

3 Industrial Reproductive Toxicology (IRDG) Computer Assisted Sperm Analysis (CASA) Group, 2000: Rat sperm morphological assessment guideline document [online]. Website http://www.irdg.co.uk/Sperm_morphology.pdf [accessed 10 May 2019].
